# Supplementary material for: Understanding the transition from paroxysmal to persistent atrial fibrillation
Source: Phys Rev Res. Author manuscript; Available in PMC 2020 Jun 30. (PMC7326608; doi:10.1103/PhysRevResearch.2.023311)
Supplement: Supplementary Material [file EMS86644-supplement-Supplementary_Material.zip › Supplementary Material/MF_Supplementary_Material.pdf]

Understanding the transition from paroxysmal to persistent atrial fibrillation from micro-anatomical re-entry in a simple model: Supplementary Material

Alberto Ciacci[1,2,4,*], Max Falkenberg[1,2,4,*], Kishan A. Manani[1,2,3],
Tim S. Evans[1,2], Nicholas S. Peters[4], Kim Christensen[1,2,4]
[1] *Blackett Laboratory, Imperial College London, London SW7 2BW, United Kingdom*
[2] *Center for Complexity Science, Imperial College London, London SW7 2AZ, United Kingdom*
[3] *National Heart and Lung Institute, Imperial College London, London W12 0NN, United Kingdom*
[4] *ElectroCardioMaths Programme, Imperial Centre for Cardiac Engineering, Imperial College London, London W12 0NN, United Kingdom*
[*] ***These authors have equally contributed to this work***
Corresponding author: kim.christensen@imperial.ac.uk
(Dated: April 22, 2020)

The material below is intended as a supplement to the manuscript "Understanding the transition from paroxysmal to persistent atrial fibrillation from micro-anatomical re-entry in a simple model" by *Ciacci et al.* The figures included show the step by step evolution of individual re-entrant circuits in the CMP model. The figures shown correspond to the structure types listed in Fig. (2), Fig. (3), Fig. (9) and Fig. (10) of the main manuscript. For circuits with complex activation dynamics, separate examples are provided for the cases where the critical structure does, and does not activate to form a re-entrant circuit. Videos of each of the circuits is available online. Captions for the videos are provided on page 2 of this document. The code used throughout this project is available at "https://github.com/AlbertoCiacci/understanding_the_transition_from_paroxysmal_to_persistent_AF".

I. VIDEOS

A. Critical Structure Diagrams

The videos for the critical structures shown in Fig. (2), Fig. (3), Fig. (9) and Fig. (10) of the main manuscript are included in the folder “Critical Structure Diagrams”. Each video corresponds to one of the figures below. Subtitles are included in each video describing the dynamics.

B. Real Critical Structures

Short animations of the CMP model are included which zoom in on the dynamics of the underlying critical structures for Fig. 5(d), Fig. 5(h) and Fig. 10 in the folder “Real Critical Structures”. Active nodes are shown in white, resting nodes in black, and refractory nodes in greyscale. Nodes which are susceptible to conduction block have a red border. The blue (black) boxes indicate the key critical structures driving AF when active (inactive).

Fig 5(d) video: Two simple re-entrant circuits are coupled such that the failure of a conduction blocking node in one circuit can be circumvented by the coupled circuit. This results in fibrillatory events which require two successive conduction blocking node failures to terminate the fibrillation.

Fig 5(h) video: A complex re-entrant circuit is shown which can be activated from a single conduction blocking node failure, but requires multiple conduction blocking node failures to terminate fibrillation. The circuit shown results in activity lasting at least 10^6 time steps.

Fig 10 video: A complex re-entrant circuit is shown which requires a large number of conduction blocking node failures to terminate, and is coupled to adjacent structures such that the activity driven by this region results in persistent AF lasting at least 10^9 time steps. In real time, this corresponds to approximately 1 month.

C. CMP AF Spectrum

Short videos of the full CMP model domain are shown for sinus rhythm, paroxysmal and persistent AF in the folder “CMP AF Spectrum”. These examples are similar to those shown in Fig. 16 of the main manuscript. Active nodes are shown in white, resting nodes in black, and refractory nodes in greyscale. For more detail see appendix C of the main manuscript.

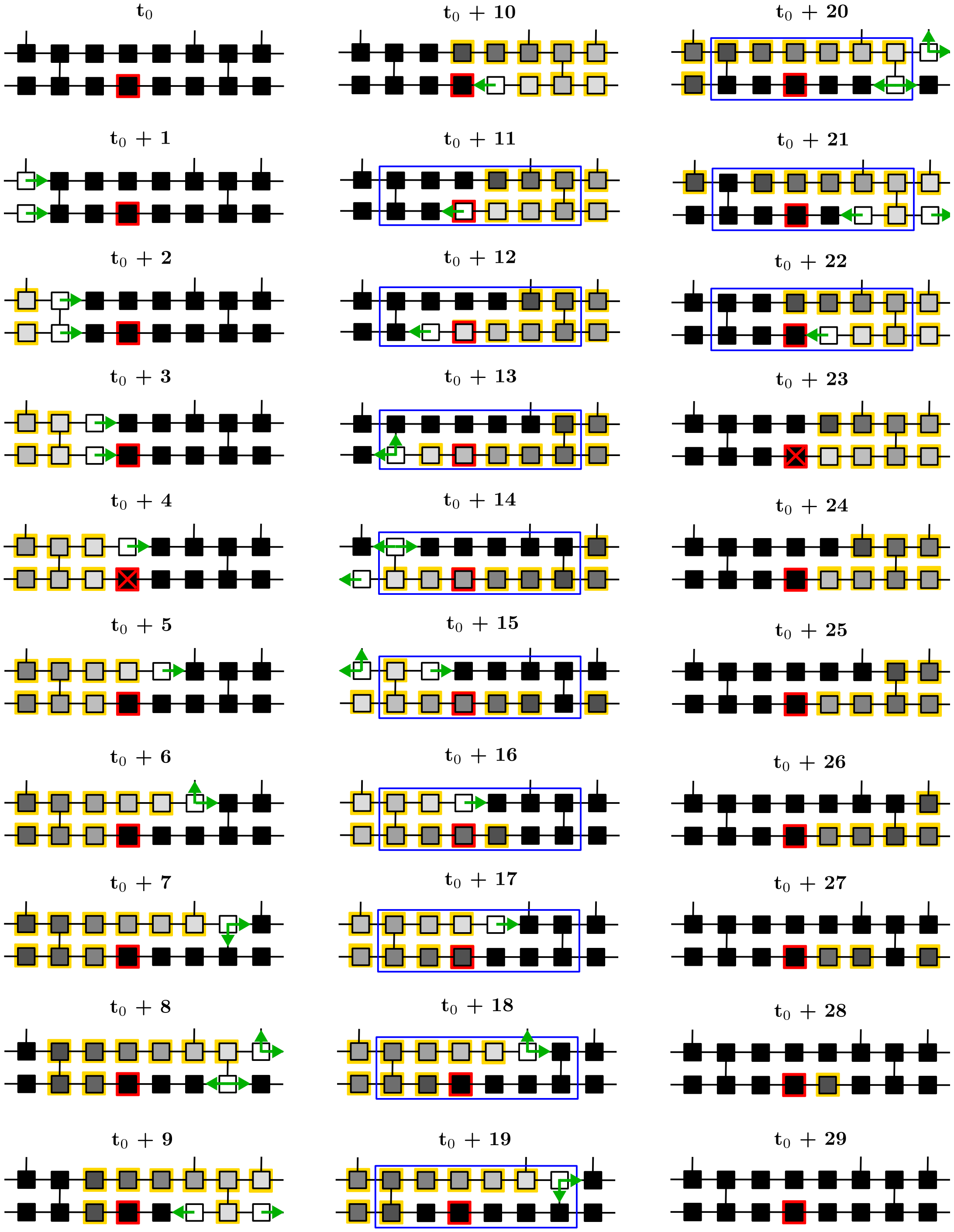

FIG. 1. Activation and deactivation of the critical structure presented in Fig. 3 (a) of the main manuscript. Resting cells are shown in black. Excited cells are shown in white. Refractory cells are shown in grey-scale with a yellow border. Green arrows indicate the movement of the excitation wavefront. Red bordered cells are susceptible to conduction block. A red cross indicates that a cell susceptible to conduction block has failed to activate when prompted to do so by a neighbour. The blue box indicates the region corresponding to an active re-entrant circuit.

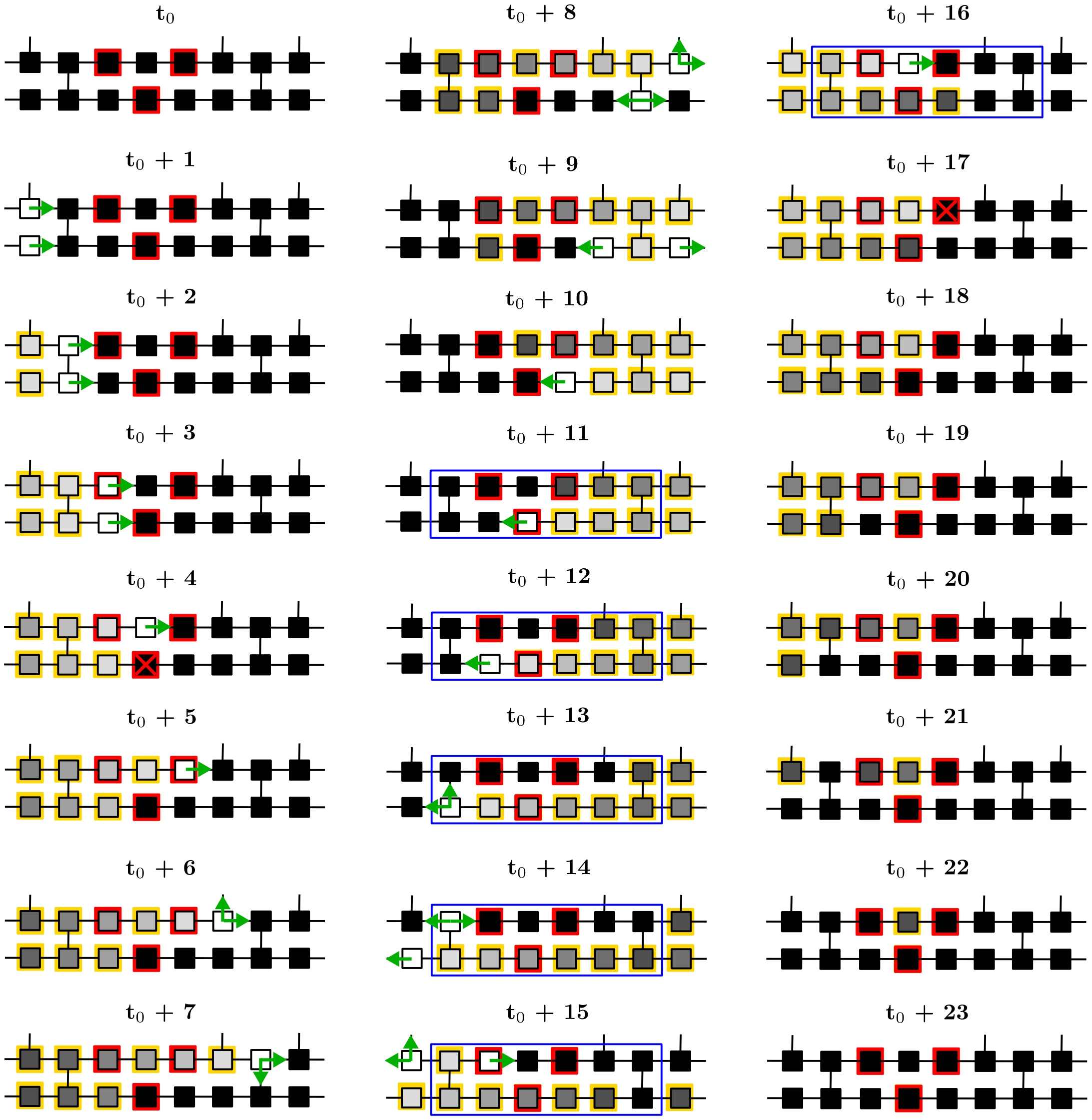

FIG. 2. Activation and deactivation of the critical structure presented in Fig. 3 (b) of the main manuscript. Resting cells are shown in black. Excited cells are shown in white. Refractory cells are shown in grey-scale with a yellow border. Green arrows indicate the movement of the excitation wavefront. Red bordered cells are susceptible to conduction block. A red cross indicates that a cell susceptible to conduction block has failed to activate when prompted to do so by a neighbour. The blue box indicates the region corresponding to an active re-entrant circuit.

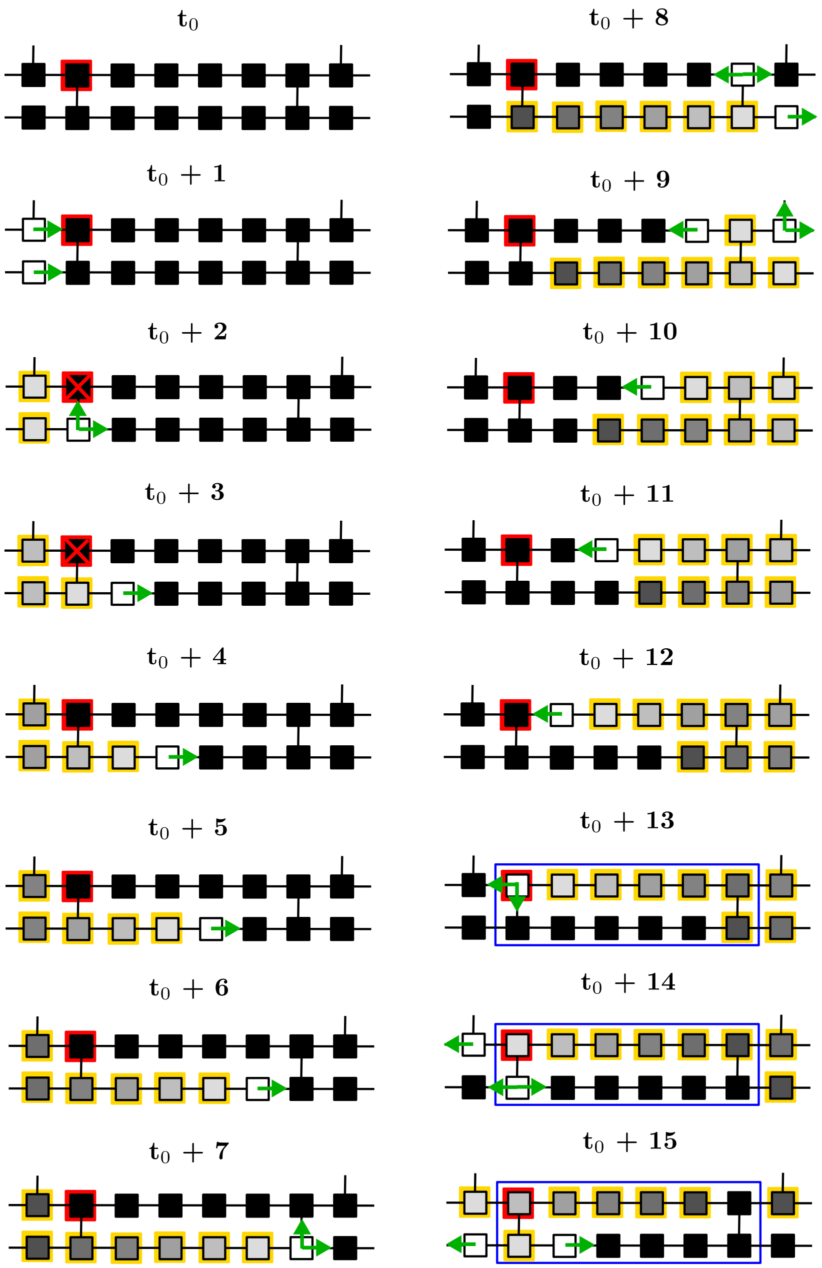

FIG. 3. Successful activation of the critical structure presented in Fig. (3) (c) of the main manuscript. Resting cells are shown in black. Excited cells are shown in white. Refractory cells are shown in grey-scale with a yellow border. Green arrows indicate the movement of the excitation wavefront. Red bordered cells are susceptible to conduction block. A red cross indicates that a cell susceptible to conduction block has failed to activate when prompted to do so by a neighbour. The blue box indicates the region corresponding to an active re-entrant circuit.

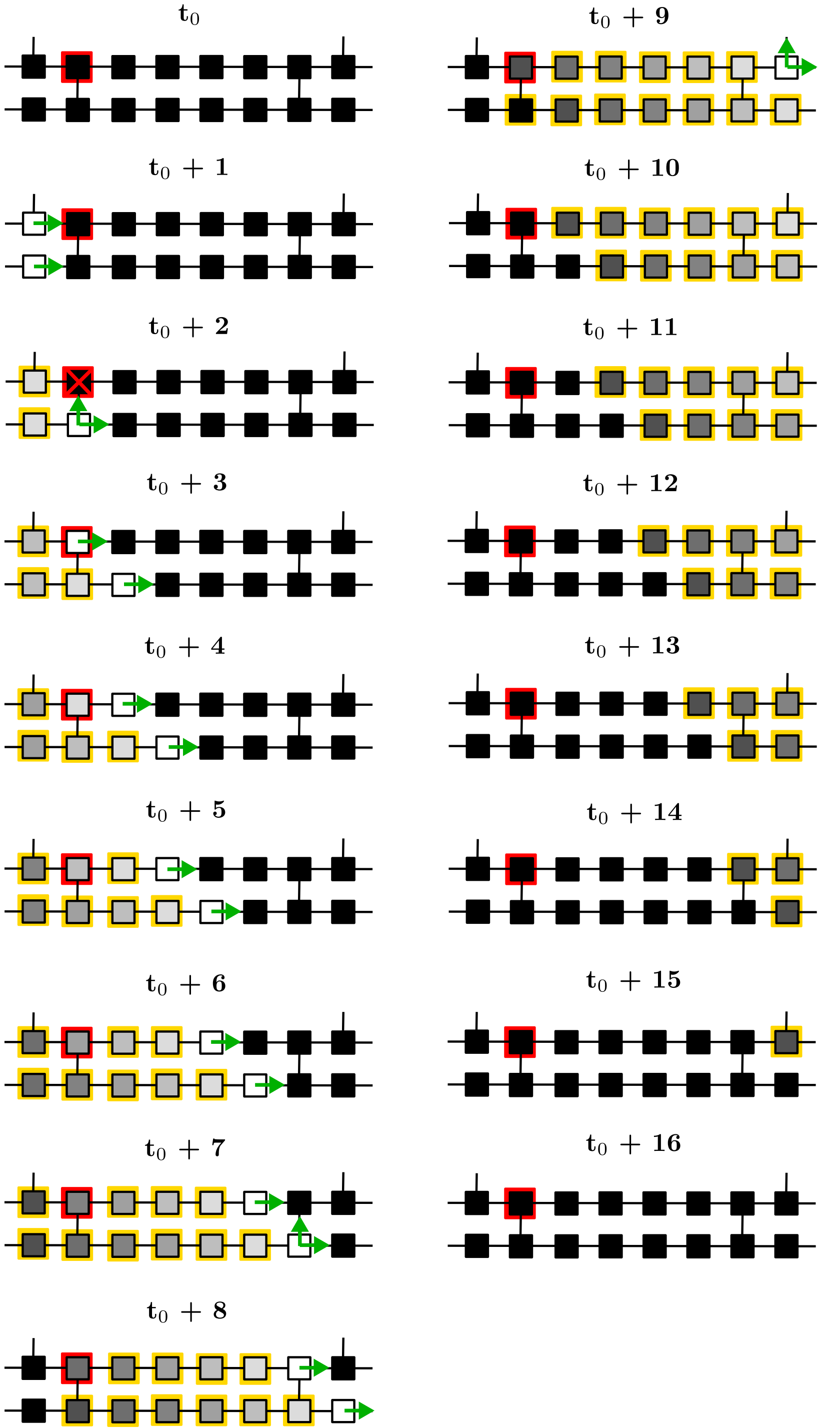

FIG. 4. Failed activation of the critical structure presented in Fig. (3) (c) of the main manuscript. Resting cells are shown in black. Excited cells are shown in white. Refractory cells are shown in grey-scale with a yellow border. Green arrows indicate the movement of the excitation wavefront. Red bordered cells are susceptible to conduction block. A red cross indicates that a cell susceptible to conduction block has failed to activate when prompted to do so by a neighbour. The blue box indicates the region corresponding to an active re-entrant circuit.

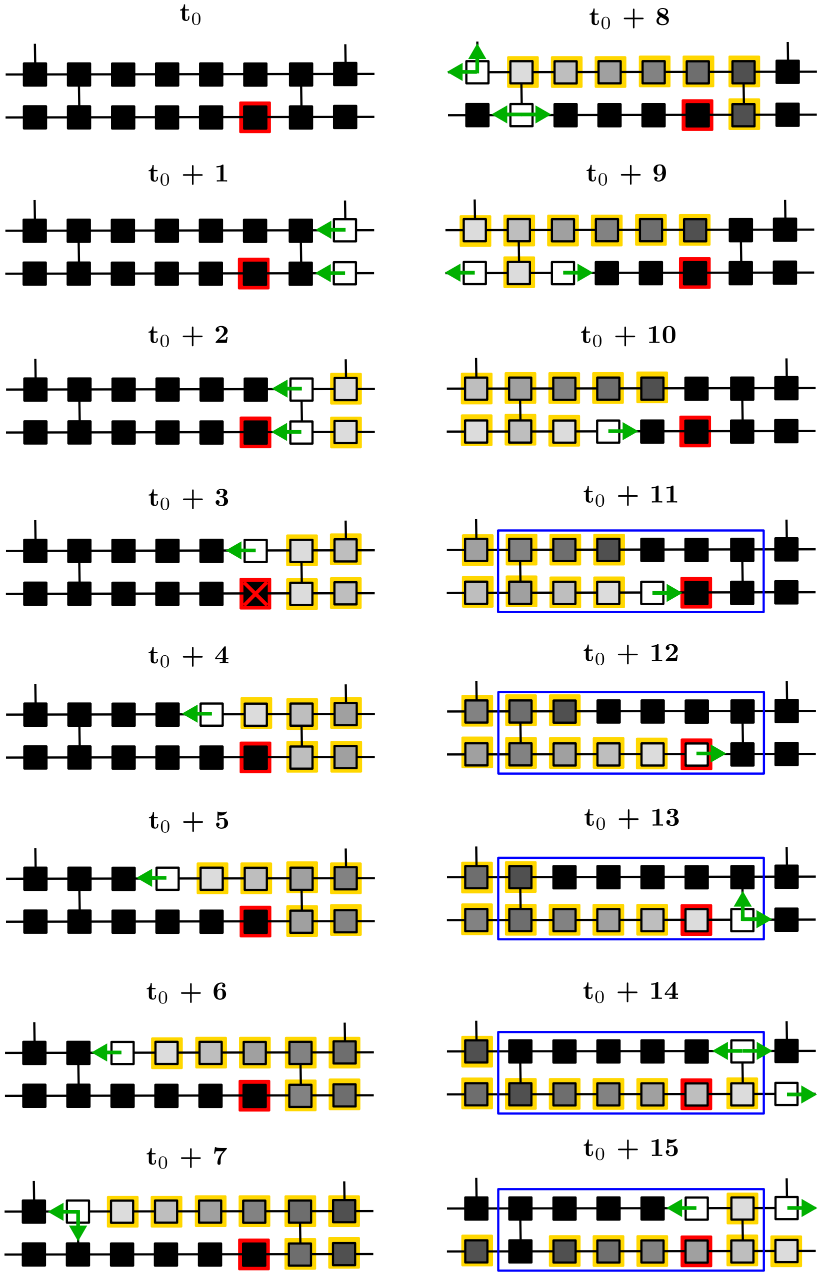

FIG. 5. Successful activation of the critical structure presented in Fig. (3) (d) of the main manuscript. Resting cells are shown in black. Excited cells are shown in white. Refractory cells are shown in grey-scale with a yellow border. Green arrows indicate the movement of the excitation wavefront. Red bordered cells are susceptible to conduction block. A red cross indicates that a cell susceptible to conduction block has failed to activate when prompted to do so by a neighbour. The blue box indicates the region corresponding to an active re-entrant circuit.

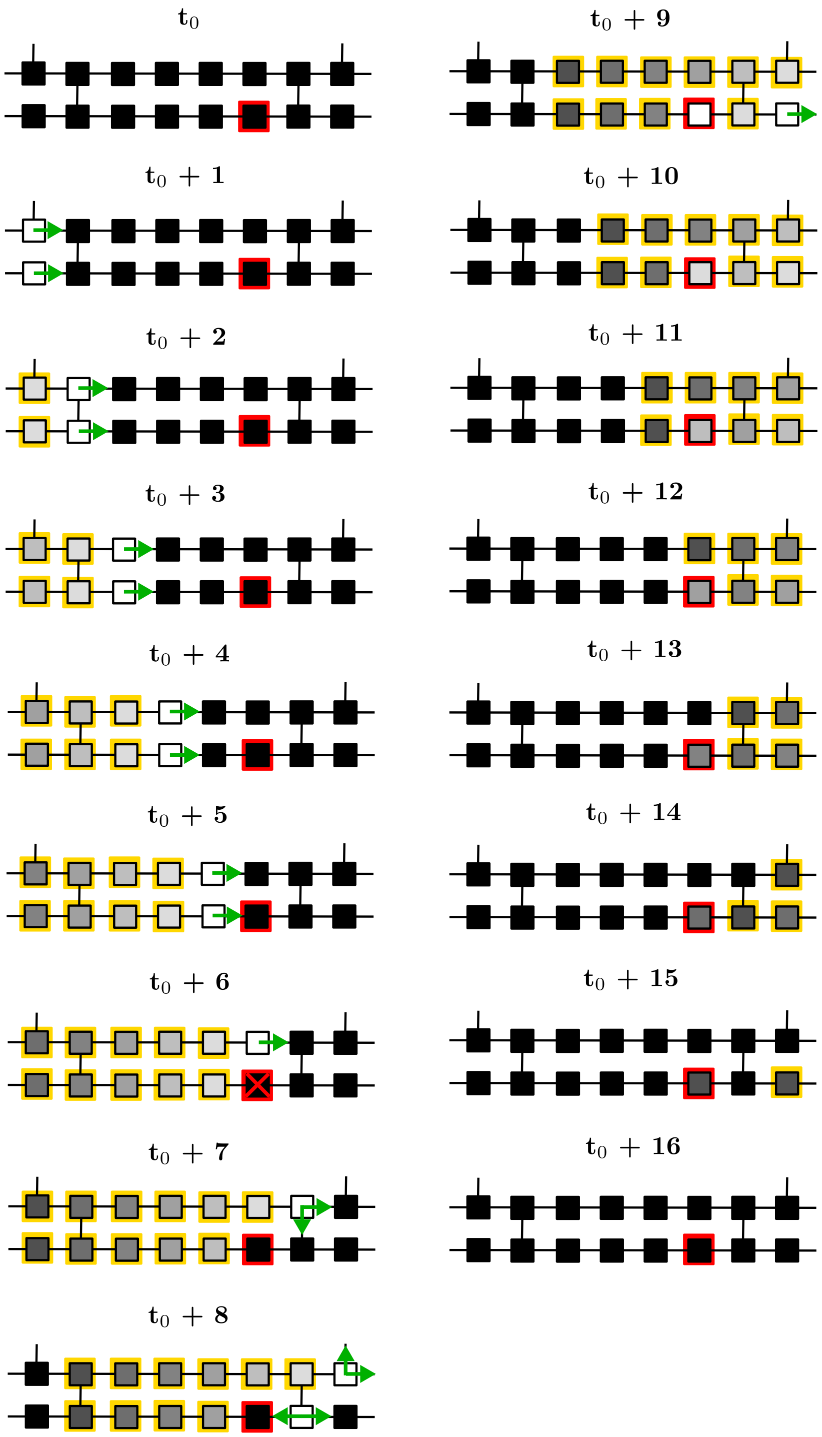

FIG. 6. Failed activation of the critical structure presented in Fig. (3) (d) of the main manuscript. Resting cells are shown in black. Excited cells are shown in white. Refractory cells are shown in grey-scale with a yellow border. Green arrows indicate the movement of the excitation wavefront. Red bordered cells are susceptible to conduction block. A red cross indicates that a cell susceptible to conduction block has failed to activate when prompted to do so by a neighbour. The blue box indicates the region corresponding to an active re-entrant circuit.

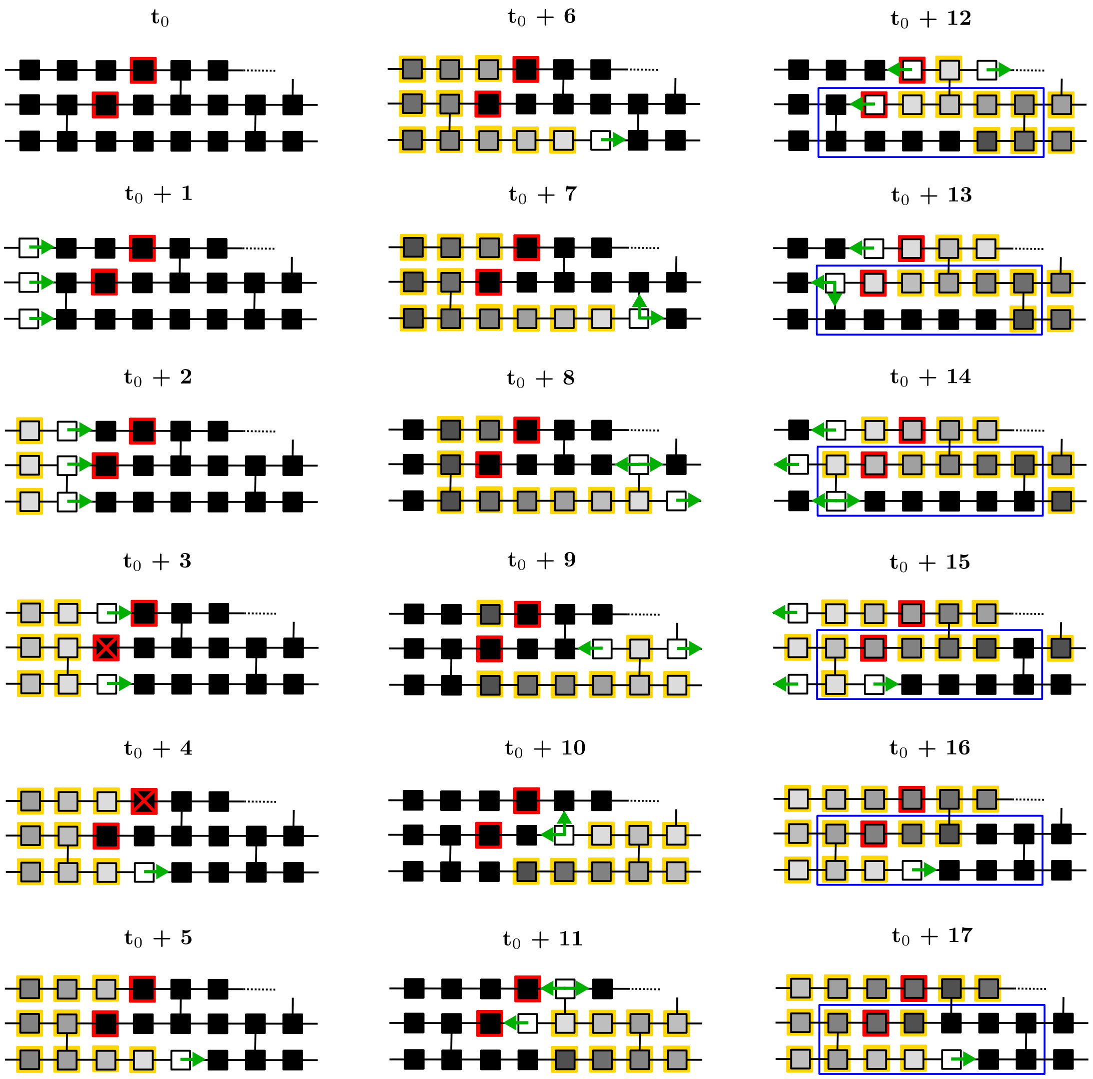

FIG. 7. Successful activation of the critical structure presented in Fig. (3) (e) of the main manuscript. Resting cells are shown in black. Excited cells are shown in white. Refractory cells are shown in grey-scale with a yellow border. Green arrows indicate the movement of the excitation wavefront. Red bordered cells are susceptible to conduction block. A red cross indicates that a cell susceptible to conduction block has failed to activate when prompted to do so by a neighbour. The blue box indicates the region corresponding to an active re-entrant circuit.

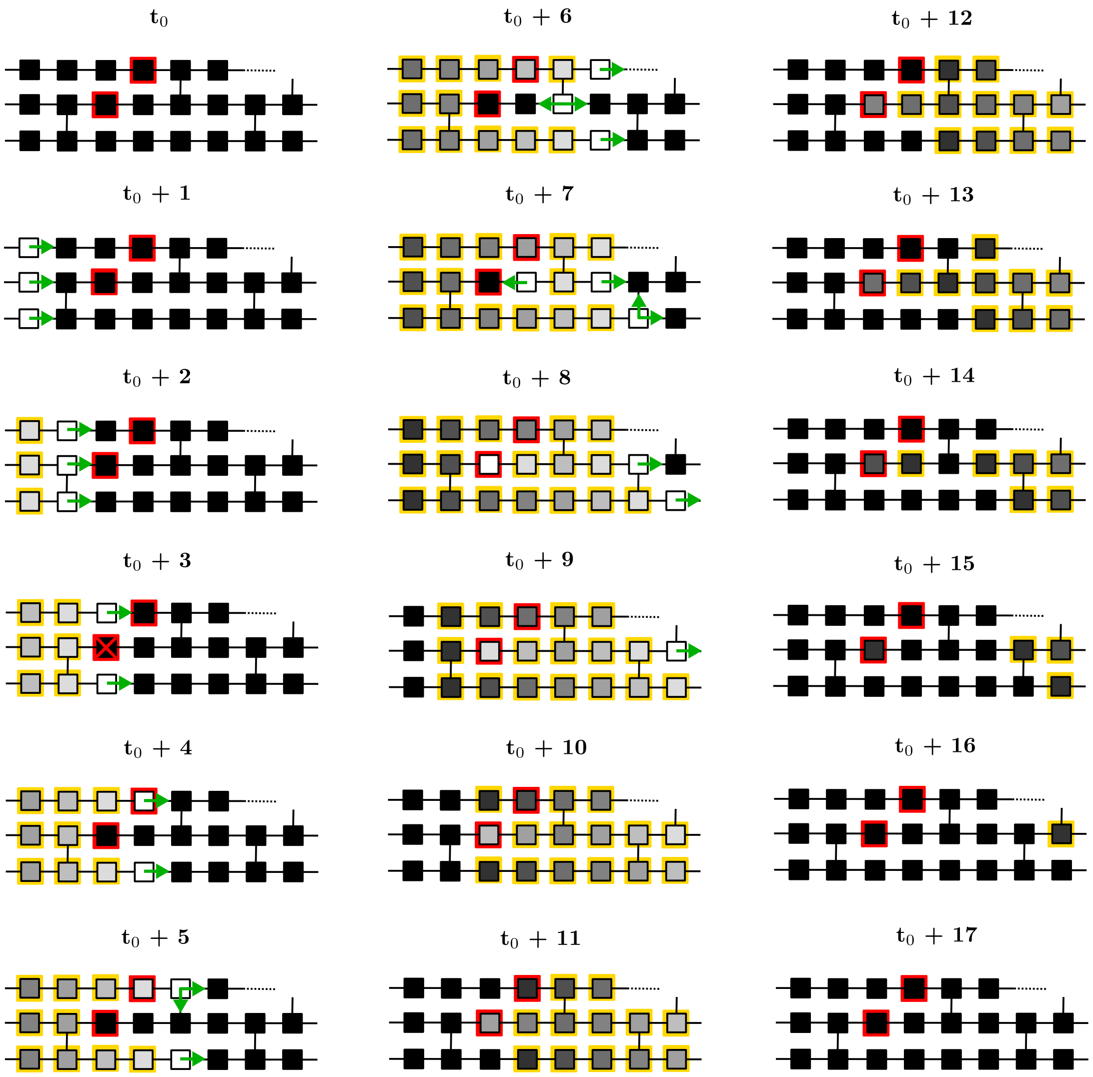

FIG. 8. Failed activation of the critical structure presented in Fig. (3) (e) of the main manuscript. Resting cells are shown in black. Excited cells are shown in white. Refractory cells are shown in grey-scale with a yellow border. Green arrows indicate the movement of the excitation wavefront. Red bordered cells are susceptible to conduction block. A red cross indicates that a cell susceptible to conduction block has failed to activate when prompted to do so by a neighbour. The blue box indicates the region corresponding to an active re-entrant circuit.

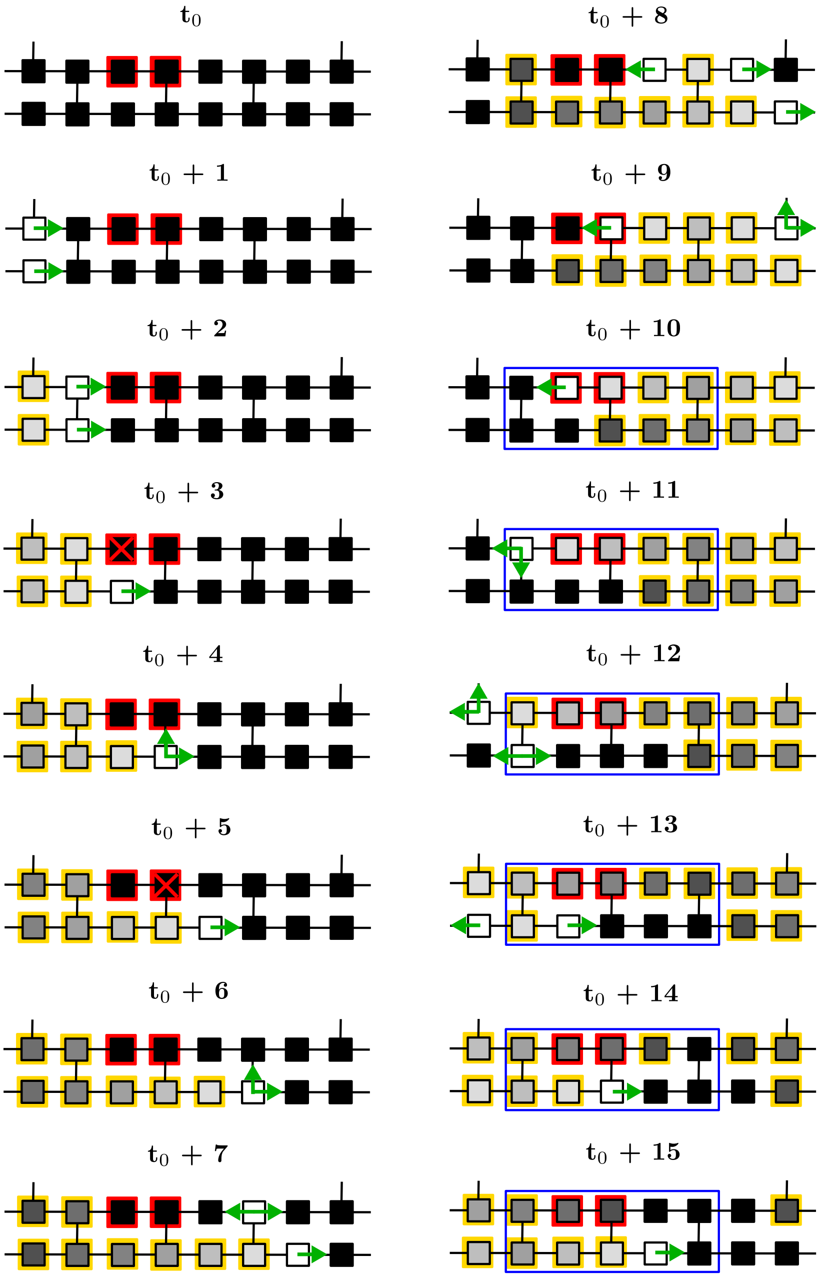

FIG. 9. Successful activation of the critical structure presented in Fig. (3) (f) of the main manuscript. Resting cells are shown in black. Excited cells are shown in white. Refractory cells are shown in grey-scale with a yellow border. Green arrows indicate the movement of the excitation wavefront. Red bordered cells are susceptible to conduction block. A red cross indicates that a cell susceptible to conduction block has failed to activate when prompted to do so by a neighbour. The blue box indicates the region corresponding to an active re-entrant circuit.

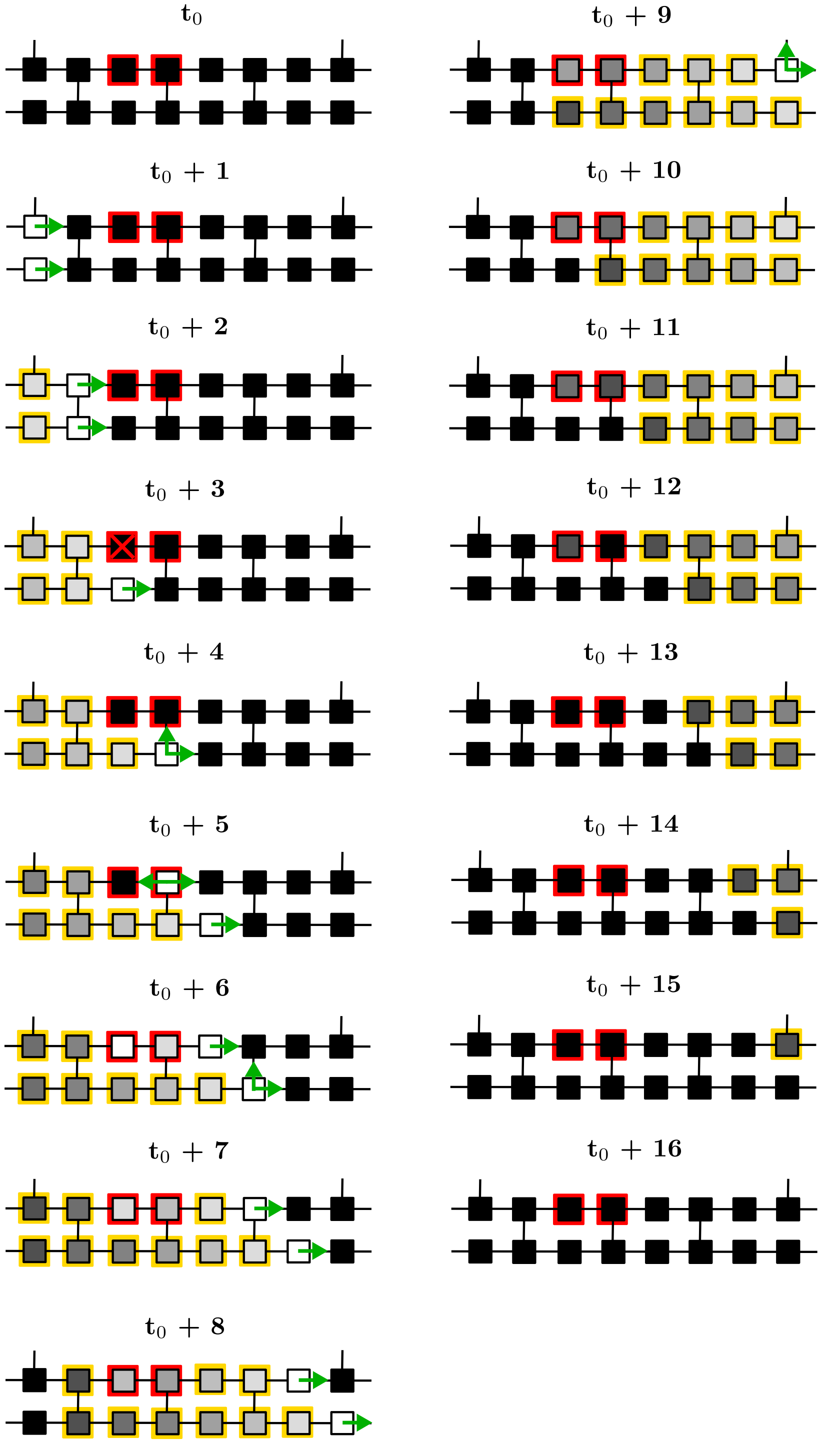

FIG. 10. Failed activation of the critical structure presented in Fig. (3) (f) of the main manuscript. Resting cells are shown in black. Excited cells are shown in white. Refractory cells are shown in grey-scale with a yellow border. Green arrows indicate the movement of the excitation wavefront. Red bordered cells are susceptible to conduction block. A red cross indicates that a cell susceptible to conduction block has failed to activate when prompted to do so by a neighbour. The blue box indicates the region corresponding to an active re-entrant circuit.

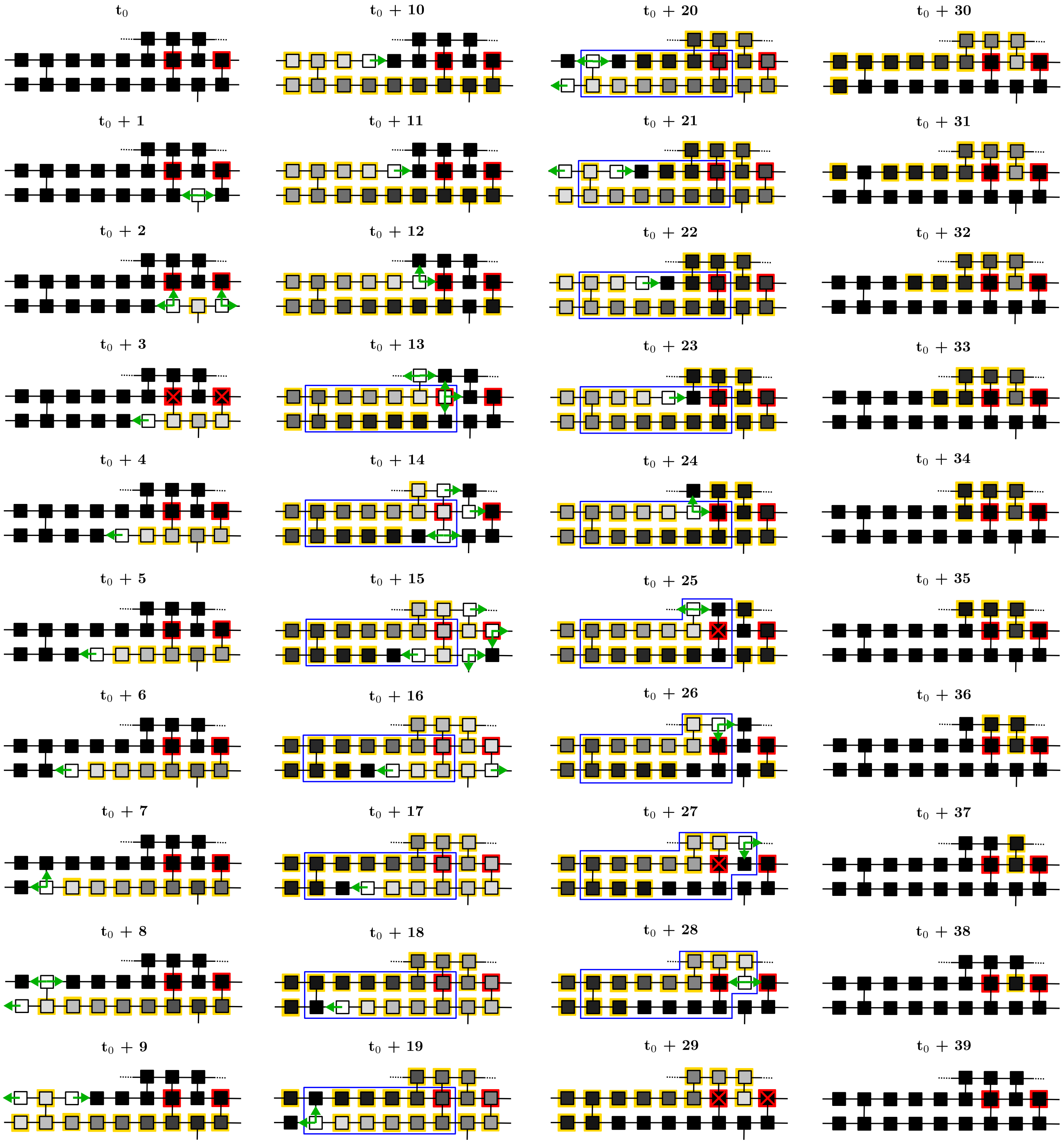

FIG. 11. Activation and deactivation of the complex critical structure presented in Fig. (9) of the main manuscript. Resting cells are shown in black. Excited cells are shown in white. Refractory cells are shown in grey-scale with a yellow border. Green arrows indicate the movement of the excitation wavefront. Red bordered cells are susceptible to conduction block. A red cross indicates that a cell susceptible to conduction block has failed to activate when prompted to do so by a neighbour. The blue box indicates the region corresponding to an active re-entrant circuit.

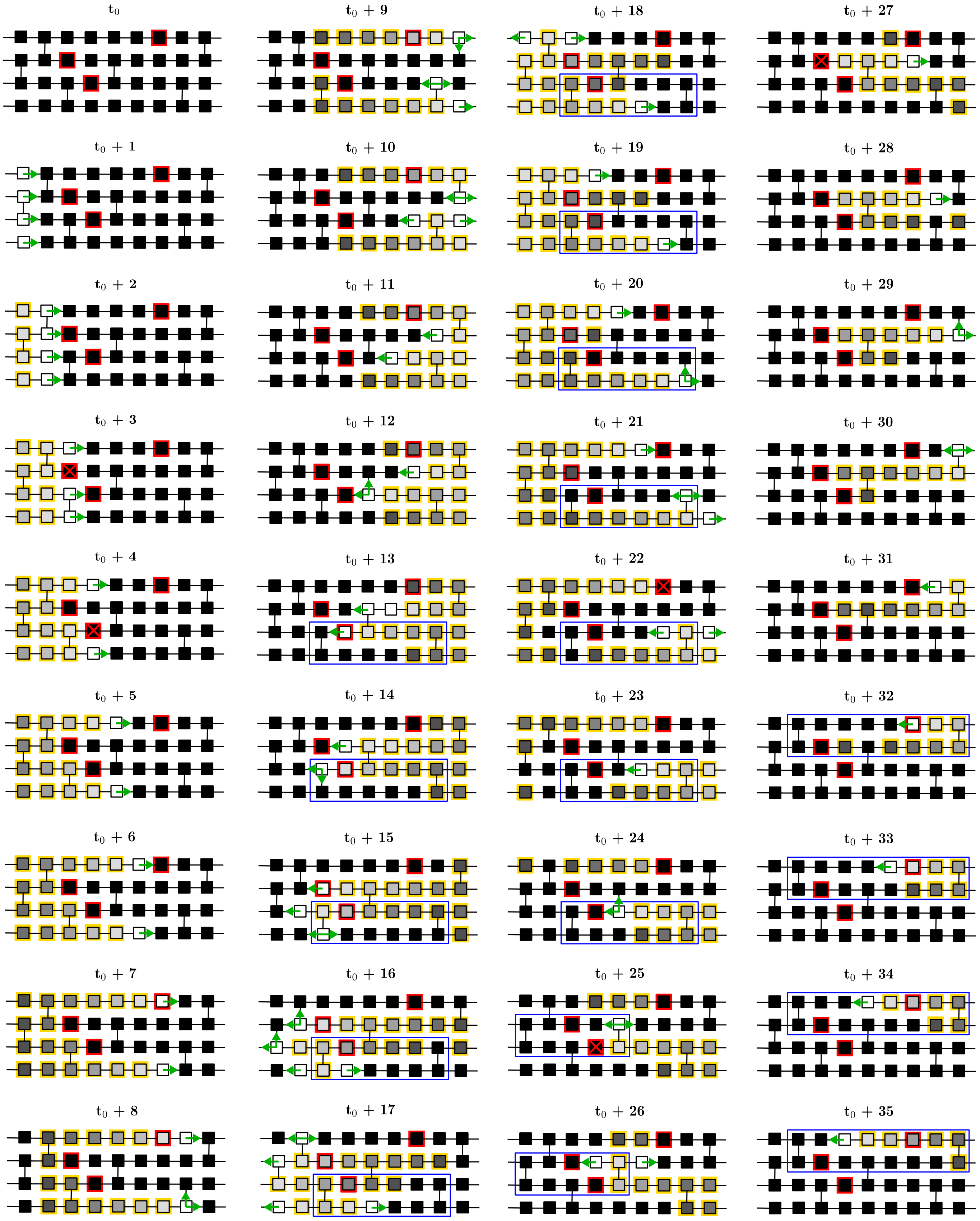

FIG. 12. Sequence of activations of the coupled re-entrant circuits presented in Fig. (10) of the main manuscript. Resting cells are shown in black. Excited cells are shown in white. Refractory cells are shown in grey-scale with a yellow border. Green arrows indicate the movement of the excitation wavefront. Red bordered cells are susceptible to conduction block. A red cross indicates that a cell susceptible to conduction block has failed to activate when prompted to do so by a neighbour. The blue box indicates the region corresponding to an active re-entrant circuit.
