# Supplementary material for: Understanding the transition from paroxysmal to persistent atrial fibrillation
Source: Phys Rev Res. Author manuscript; Available in PMC 2020 Jun 30. (PMC7326608; doi:10.1103/PhysRevResearch.2.023311)
Supplement: Appendices [file EMS86644-supplement-Appendices.pdf]

APPENDIX A: MEAN-FIELD MODEL OF AF IN CONTINUOUS TIME

The MF model can be extended to the continuous time case (cMF), providing us with a framework in which the time in fibrillation can be computed analytically. Let $\tilde{p}(k,t)$ be the probability of observing k active simple reentrant circuits at time t. When the interval between two consecutive time steps Δt is sufficiently small (i.e., $\Delta t \to 0$), we have at most one event (activation or de-activation) per interval. In these settings, the dynamics of $\tilde{p}(k,t)$ are described by the following master equation:

$$\frac{d\tilde{p}(k,t)}{dt} = p(N-k+1)\tilde{p}(k-1,t) - p(N-k)\tilde{p}(k,t) + q(k+1)\tilde{p}(k+1,t) - qk\tilde{p}(k,t) \quad \text{for } k > 1, \tag{A1a}$$

where the first two terms are associated with an activation process $k-1 \mapsto k$ and $k \mapsto k+1$ transitions, respectively, while the last two are associated with $k+1 \mapsto k$ and $k \mapsto k-1$ transitions, respectively. Because the activation rate is different when the system has no active particles, see Eq. (6a), we need to take special care of the $k=1$ and $k=0$ cases. If the term p_0 represents the activation rate when the system has no active particles, then

$$\frac{d\tilde{p}(1,t)}{dt} = p_0 N\tilde{p}(0,t) - p(N-1)\tilde{p}(1,t) + 2q\tilde{p}(2,t) - q\tilde{p}(1,t), \quad \text{for } k=1, \tag{A1b}$$

$$\frac{d\tilde{p}(0,t)}{dt} = -p_0 N\tilde{p}(0,t) + q\tilde{p}(1,t), \quad \text{for } k=0. \tag{A1c}$$

TABLE I. Time in AF in the cMF model for different combinations of p/p_0 and N. We observe that for small N the time in AF is significantly higher when the ratio p/p_0 is small. These differences vanish as N increases.

N	0	1	2	3	4	5	10	∞
$p/p_0 = 4.4$	0	0.185	0.405	0.614	0.773	0.876	0.996	1
$p/p_0 = 1$	0	0.5	0.75	0.875	0.938	0.969	0.999	1

We enforce the boundary conditions that $\tilde{p}(k,t) = 0$ for $k < 0$ and $k > N$. We will find the steady state solution $\tilde{p}(k) = \lim_{t\to\infty} \tilde{p}(k,t)$ where the derivatives on the left-hand side of Eq. (A1) are zero by the ansatz

$$\tilde{p}(k) = \begin{cases} A\binom{N}{k}\left(\frac{q}{p}\right)^{N-k} + B\delta_{k,0} & \text{for } k = 0, 1, \dots, N, \\ 0 & \text{for } k < 0 \text{ or } k > N, \end{cases} \tag{A2}$$

where $\delta_{i,j}$ is the Kronecker δ function. By inserting the ansatz into Eq. (A1a), we confirm that it solves the steady state equation for $k > 1$. However, in our case it simplifies further as $p = q = \epsilon/\langle \ell \rangle$, see Eq. (6a), so $q/p = 1$.

We can determine the two constants A and B by requiring that Eq. (A2) solves Eqs. (A1b)–(A1c) together with the normalization constraint: inserting the ansatz into Eq. (A1c), recalling $p = q$, we find

$$0 = -p_0 AN - p_0 NB + qAN, \tag{A3}$$

implying that

$$B = A\left(\frac{p}{p_0} - 1\right). \tag{A4a}$$

Note that $p = p_0 \Rightarrow B = 0$, that is, $\tilde{p}(0)$ does not have a special status but when $p \neq p_0 \Rightarrow B \neq 0$, and B is an extra contribution to $\tilde{p}(0)$; see Eq. (A2). We now require normalization, that is,

$$1 = \sum_{k=0}^{N} \tilde{p}(k) = A2^N + B. \tag{A4b}$$

Solving Eqs. (A4) for A and B we find

$$A = \frac{1}{2^N + p/p_0 - 1}, \tag{A5a}$$

$$B = \frac{p/p_0 - 1}{2^N + p/p_0 - 1}, \tag{A5b}$$

yielding

$$\tilde{p}(0) = \frac{p/p_0}{2^N + p/p_0 - 1}. \tag{A6}$$

Having obtained the analytical solutions, the fraction of time the system spends in AF for the cMF model is given by

$$1 - \tilde{p}(0) = \frac{2^N - 1}{2^N + p/p_0 - 1}. \tag{A7}$$

The time in AF for the cMF model is shown in Fig. 13. It is interesting to contrast this result with a simple birth-death process where $p = p_0$. The time in AF is shown for the simple

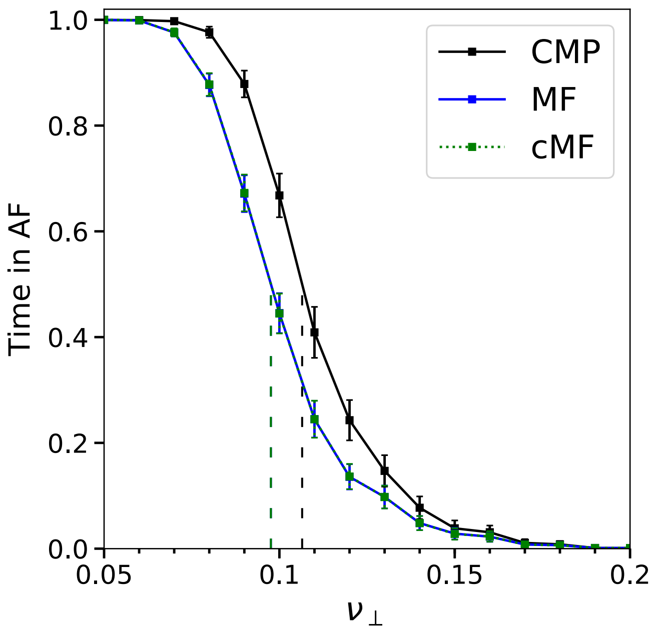

FIG. 13. Phase diagram of the time in AF as a function of the fraction of transversal connections $\nu_\perp$ for the CMP (black), the MF (blue) and the cMF (green) models. We use the parameters of the CMP model (i.e., N, ϵ, and $\langle \ell \rangle$) to calibrate the cMF model and calculate the time in AF according to Eq. (A7). The phase diagrams for the MF and the cMF models are perfectly compatible. Both models significantly underestimate the time in AF with respect to the CMP model. Sharp transitions in the time in AF occur around the threshold values $\nu_\perp^* \approx 0.11$ (CMP, black dashed line), $\nu_\perp^* \approx 0.10$ (MF, blue dashed line), and $\nu_\perp^* \approx 0.10$ (cMF, green dashed line).

birth-death process and for the cMF calibrated to the CMP model, $p/p_0 = T/\tau = 4.4$, in Table I. The results indicate that for $N = 0$, neither model enters AF. As N is increased, the time in AF is initially much higher in the birth-death process than the cMF, but this difference vanishes as N becomes large. Only when $N \to \infty$ does the model spend 100% of the time in AF. Since N must be finite in the CMP model, this indicates that the cMF cannot explain persistent AF.

APPENDIX B: ENHANCED MEAN-FIELD MODEL OF AF

The phase diagrams discussed in Fig. 7 reveal significant differences between the CMP and MF models as the latter underestimates the time in AF. One may assert that this discrepancy stems from a poor replication of the interactions between reentrant circuits, and in particular from the exclusion of the nonspatial features of critical structures (e.g., particles are assumed to have the same length) from the MF model. In this section, we provide further evidence against this hypothesis by showing that modeling various nonspatial features of critical structures does not mitigate the differences between the CMP and MF models. To do so, we introduce an enhanced version of the MF model (eMF) in which each particle retains the length of the associated critical structure and changes its state at specific time steps, depending on the overall configuration of the system. The purpose of the eMF is to indicate that the nonspatial simplifications in the MF model are not responsible for the discrepancy in the time in AF between the MF model and the CMP model.

In the eMF model, the system is represented by the state vector $P(t) = (p_1(t), \dots, p_N(t))$, where $p_j(t) \in \{0, 1\}$, $j = 1, \dots, N$ is the state of the jth particle at time t and N is the number of particles corresponding to the simple critical structures found across the CMP lattice. When $p_j(t) = 1$ ($p_j(t) = 0$), the jth particle is active (inactive) at time t. The number of active particles at time t is

$$N_a(t) = \sum_{j=1}^{N} p_j(t). \tag{B1}$$

In line with the original MF model, the system is in sinus rhythm when $N_a(t) = 0$ and in AF when $N_a(t) > 0$. The jth particle $p_j(t)$ can change its state at a specific time t_j^*. We set the first switching time for each particle as $t_j^* \to U_j$, where U_j, $j = 1, \dots, N$ is a uniformly distributed integer random variable in $[1, L]$. This mimics the first planar wave front released from the pacemaker reaching critical regions at different time steps due to their different locations. As soon as the simulation time t matches t_j^*, the jth particle changes its state with probability ϵ. Independently of whether the jth particle has changed its state or not, its next switching time t_j^* is updated,

$$t_j^* \to \begin{cases} t_j^* + \min\limits_{p:p(t+1)=1} \ell_p, & \text{if } N_a(t+1) > 0 \text{ and } p_j(t+1) = 0, \\ t_j^* + \ell_j, & \text{if } N_a(t+1) > 0 \text{ and } p_j(t+1) = 1, \\ t_j^* + T, & \text{if } N_a(t+1) = 0, \end{cases} \tag{B2}$$

where $t + 1$ indicates that the update is based on the characteristics of the system observed immediately after the eventual state change of the jth particle. When $N_a(t + 1) = 0$, the jth particle will attempt to switch its state in T time steps. This mimics sinus rhythm in the CMP model where the planar wave front released from the sinus nodes reaches a critical structure every T time steps. When $N_a(t + 1) > 0$, particles try to switch their states more frequently. This reflects the intense activity (e.g., number of active nodes per time step) observed in AF episodes occurring in the CMP lattice.

In the eMF model, the length of the shortest particle dictates the period between two consecutive attempts to activate a dormant region. For instance, the jth particle that turns (or remains) off at time $t_j^* = t$ will attempt to activate again at time

$$t_j^* = t_j^* + \min_{p:p(t+1)=1} \ell_p, \tag{B3}$$

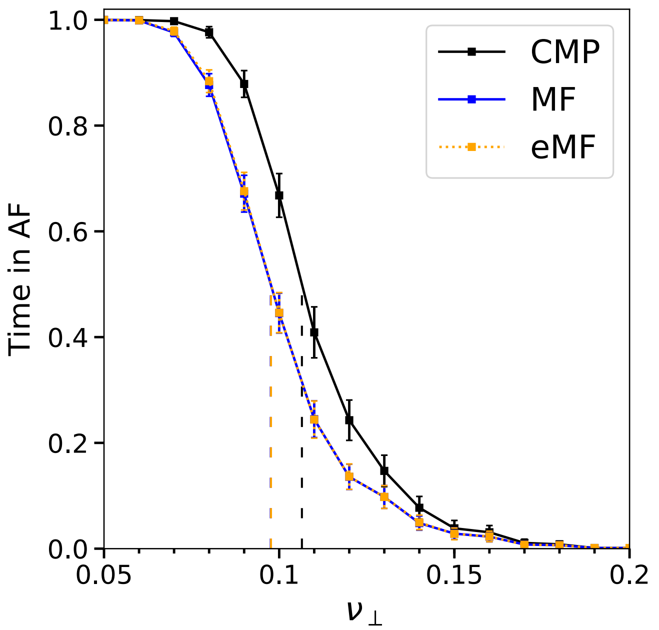

FIG. 14. Phase diagram of the time in AF as a function of the fraction of transversal connections $\nu_\perp$ for the CMP (black), MF (blue), and eMF (orange) models. We use the parameters of the CMP model (i.e., N, ϵ, and $\langle \ell \rangle$) and the lengths of the tracked simple critical structures (i.e., $\ell_{p_1}, \dots, \ell_{p_N}$) to calibrate the MF and eMF models. The phase diagrams of the MF and eMF models are perfectly compatible. Both models significantly underestimate the time in AF with respect to the CMP model. These results suggest that the spatial structure of the CMP model is responsible for the excess time the CMP model spends in AF compared to the MF model, and not the nonspatial simplifications of the MF model. Sharp transitions in the time in AF occur around the threshold values $\nu_\perp^* \approx 0.11$ (CMP, black dashed line), $\nu_\perp^* \approx 0.10$ (MF, blue dashed line; eMF, orange dashed line).

where the final term is the length of the shortest active particle at time $t + 1$. This mimics the fact that in the CMP model the length of a reentrant circuit determines the frequency at which nodes forming the hosting critical structure emit waves.

The eMF model enhances the replication of the interactions between simple critical structures by capturing potentially important spatial features that have been excluded from the original simplified MF model. The goal of this framework is to assess the contribution of the nonspatial features of critical structures to the significant discrepancies between the CMP and the MF models, see Fig. 7. We find that the phase diagram of the time in AF in the eMF model is perfectly compatible with the one derived from the MF model; see Fig. 14. This suggests that adding further layers of complexity to capture every feature of the interactions between simple critical structures is unlikely to reconcile the statistics obtained from the CMP and the MF models.

APPENDIX C: CMP MODEL DEFINITION OF AF

The CMP model with $T = 220$, $L = 200$, $\tau = 50$ is defined to be in AF when the number of active nodes in the model exceeds $1.1L$ (220 nodes). This is a working definition of AF in the CMP model and is not a unique choice. We have previously tested that this definition of AF correlates well with what would be expected from a clinical definition of AF [38]. This is shown in Fig. 15, where we plot (a) the number of active nodes in the model over time, (b) the corresponding simulated electrograms, and (c) the classification of whether the model is in AF or not according to our working definition of AF in the CMP model. The figure shows that the number of active nodes during sinus rhythm follows a regular pattern with only small scale noise around the average number of active nodes. This average falls below the practical definition of AF where we require more than 220 active nodes. The corresponding electrograms are regular and consistent with sinus rhythm pacing. When a reentrant circuit forms, the number of active nodes rapidly exceeds the threshold, and rapid, irregular activity is observed in the electrograms. The activation frequency observed is significantly higher than expected in sinus rhythm. This state is classified as being in AF according to our working definition. For more details see Ref. [38].

Note, we do not explicitly distinguish between atrial tachycardia (AT) and AF. The dynamics in the CMP model are solely based on the formation of reentrant circuits. These circuits are generally transient and are short lived. In practice, we observe regular rapid pacing in the CMP model when only a single reentrant circuit has formed. Conversely, rapid irregular pacing is observed when more than one reentrant circuit forms.

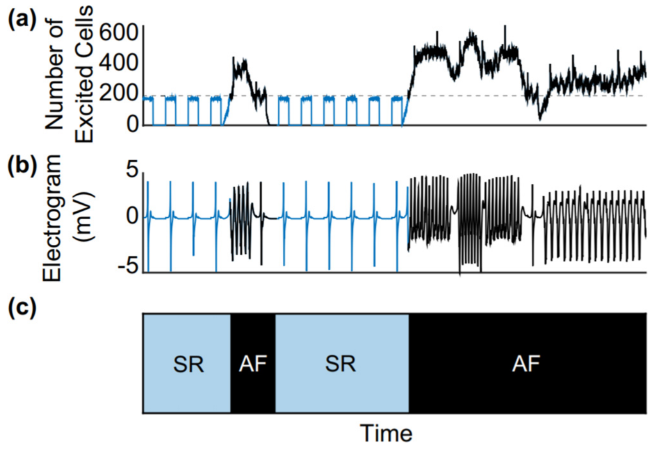

FIG. 15. The classification of AF in the CMP model. (a) The number of active nodes over time. The dashed line indicates the AF threshold of 220 active nodes. (b) Simulated electrograms derived from the CMP simulations. (c) The classification of whether the model is in sinus rhythm (SR) or AF over time. Blue lines indicate the model is in sinus rhythm, black lines indicate the model is in AF. This figure has been used with permission from Ref. [38].

APPENDIX D: ACTIVATION PATTERNS IN THE CMP MODEL

The CMP model is a highly simplified, physics style model of AF, focusing on the initiation and maintenance of microanatomical reentrant circuits. The CMP model does not consider the maintenance of AF from rotors (spiral waves). As a result, the macroscopic activation patterns in the CMP model

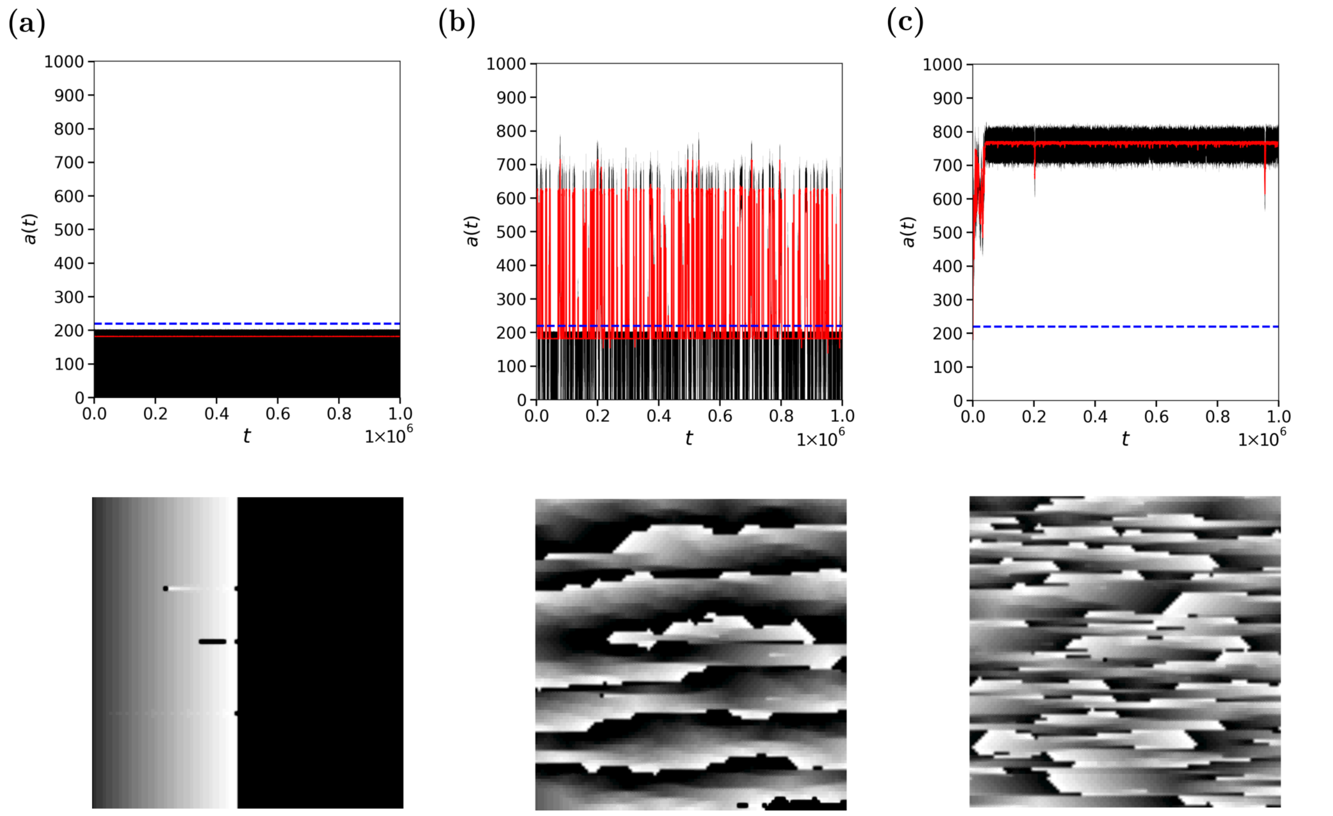

FIG. 16. The number of active cells per time step, $a(t)$; top row, and the corresponding activation patterns observed in a 100 × 100 snapshot of the CMP model, bottom row, in (a) sinus rhythm, (b) paroxysmal AF, and (c) persistent AF. Active nodes are shown in white, refractory in grayscale, and resting in black.

do not directly reflect what might be observed clinically or in other reaction-diffusion models of fibrillation.

Figure 16 shows typical activation patterns observed in the CMP model and the corresponding trace of the number of active cells. As expected, in sinus rhythm, the number of active cells is constant and falls below the threshold for AF. When the number of active cells exceeds the AF threshold, we can observe a number of different AF phenotypes in the CMP model from paroxysmal to persistent AF. Qualitatively, the activation patterns in paroxysmal and persistent AF do not show major differences, although persistent AF is typically associated with a higher dominant frequency of activation. Interestingly, there is some clinical evidence to suggest that increased dominant frequency predicts an increase in the persistence of AF [66].

In Fig. 5(h), an episode of persistent AF is shown where the number of active cells is stable over time. The simulation was generated at $\nu_\perp = 0.11$, where the average number of simple structures in the CMP model is $N < 2$; see Appendix E. This suggests that the example shown in Fig. 5(h) could plausibly be the result of one single stable reentrant circuit (although not a simple one). As a result, this example may be better thought of as a persistent episode of AT rather than AF. However, many examples of persistent AF are also observed in the CMP model where the number of active cells shows frequent fluctuations, but where the activation remains above the AF threshold; see Fig. 17.

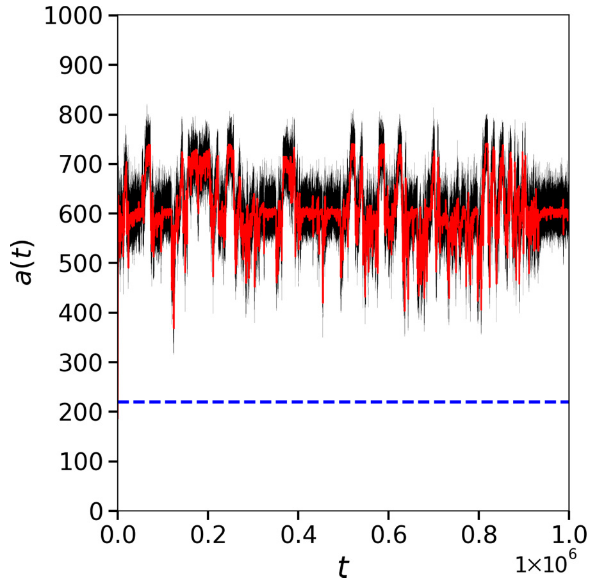

FIG. 17. An example of persistent AF in the CMP model at $\nu_\perp = 0.05$ where the number of active nodes per time step shows significant fluctuations over time. The black line indicates the raw data, with the red line indicating the moving average over a time window of $T = 220$ time steps. The blue dashed line indicates the threshold above which the CMP model is said to be in AF.

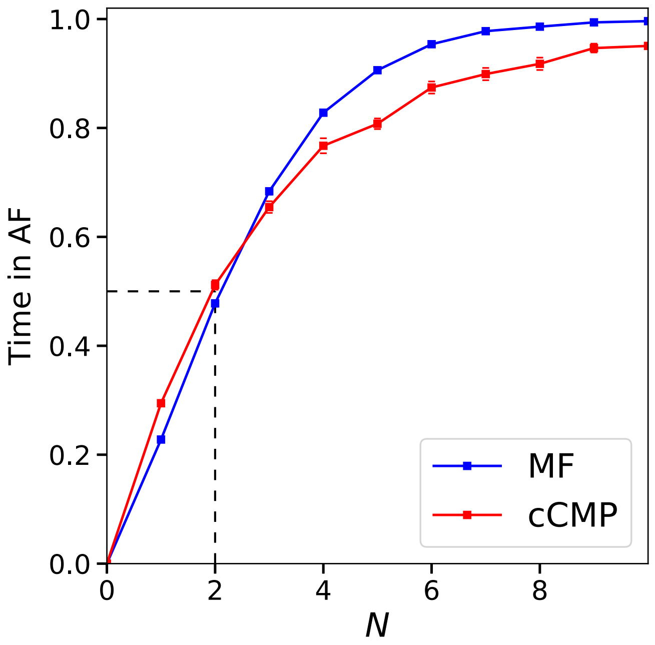

FIG. 18. The average time in AF for the cCMP (red) and MF (blue) models as a function of (1) the number of tracked simple critical structures N in the cCMP model or (2) the MF model with the corresponding number of particles.

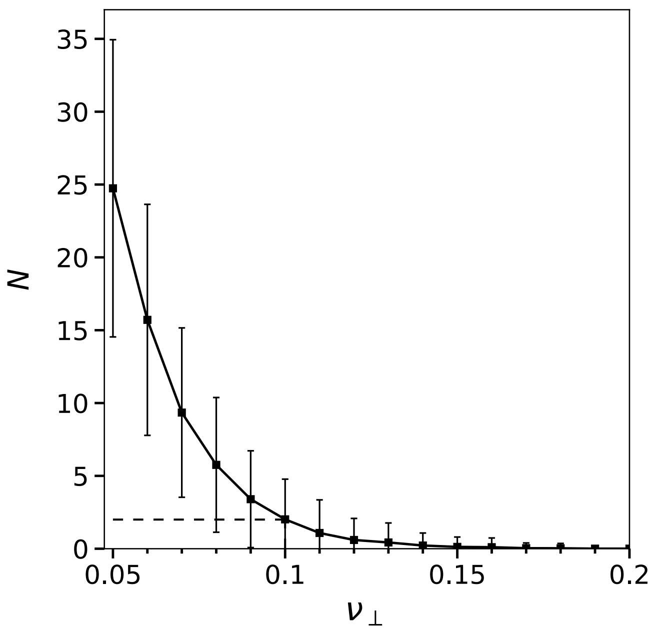

FIG. 19. The average number of tracked simple critical structures N as a function of the overall coupling value $\nu_\perp$ in the cCMP model. The errors bars indicate the 95% confidence interval calculated over 50 simulations.

APPENDIX E: CORRESPONDENCE BETWEEN THE NUMBER OF SIMPLE REENTRANT CIRCUITS AND OVERALL COUPLING

As discussed in Sec. IV, it is possible to identify the number of simple critical structures, N, defined as regions capable of forming a simple reentrant circuit, in a given instance of the CMP model. Previously, we have presented the time in AF in the CMP model as a function of the overall coupling $\nu_\perp$.

In Fig. 18 we show the time in AF as a function of the number of critical structures, N, identified in the cCMP model where we control the placement of conduction blocking nodes. For each instance of the cCMP model, the corresponding value of N is used to generate a simulation of the MF model. Figure 18 demonstrates that at low N ($N < 2$), the time in AF in the cCMP model slightly exceeds the corresponding value of the MF model. In contrast, the converse is observed at large N ($N > 2$) where the time in AF in the MF model exceeds the corresponding value in the cCMP model. This is a consequence of the spatial elements of the cCMP model which are absent in the MF model. At low N, AF episodes last a little longer in the cCMP model than in the MF model (due to slight differences in the activation and deactivation rates of the models). Conversely, at high N, the activation (or deactivation) of a particle in the MF model is independent of any other particle in the model, whereas in the cCMP model, an active reentrant circuit can suppress the activation of other critical structures which are longer than the currently active reentrant circuit. As a result, the time in AF in the cCMP model is above (below) the MF value for low (high) N.

Figure 19 shows the average number of simple critical structures detected in an instance of the cCMP model as a function of the overall coupling, $\nu_\perp$. For Fig. 19, the error bars have been chosen to show the 95% confidence interval of possible N values at a given coupling value. Fig. 18 shows that $N \approx 2$ is the crossover value above (below) which the time in AF is larger (smaller) in the MF model than in the cCMP model. Figure 19 indicates that $N = 2$ corresponds to a coupling value of $\nu_\perp \approx 0.1$. Hence, the small difference in the time in AF shown in Fig. 8 can be understood as being a consequence of the slightly different time in AF values at fixed N indicated in Fig. 18.

Note, if $N = 1$, then a single dominant reentrant circuit drives fibrillation in the CMP model. Hence, at a simplified level, this can be thought of as a form of atrial tachycardia (AT), however, this is very rare at low coupling. When multiple drivers are competing ($N > 1$), the activity in the CMP model is better associated with AF.

APPENDIX F: VIDEOS

Videos of the different structure types discussed throughout this paper are provided in the Supplemental Material with appropriate captions [44].
